# Cerebral tissue pO_2_ response to treadmill exercise in awake mice

**DOI:** 10.1038/s41598-020-70413-3

**Published:** 2020-08-07

**Authors:** Mohammad Moeini, Christophe Cloutier-Tremblay, Xuecong Lu, Ashok Kakkar, Frédéric Lesage

**Affiliations:** 1 Department of Biomedical Engineering, Amirkabir University of Technology (Tehran Polytechnic), Tehran, Iran; 2 Research Center of Montreal Heart Institute, Montréal, QC Canada; 3 Biomedical Engineering Institute, École Polytechnique de Montréal, Succursale Centre-ville, P.O. Box 6079, Montréal, QC H3C 3A7 Canada; 4 Department of Chemistry, McGill University, Montréal, QC Canada

**Keywords:** Neuroscience, Circulation, Fluorescence imaging, Molecular imaging, Optical imaging

## Abstract

We exploited two-photon microscopy and Doppler optical coherence tomography to examine the cerebral blood flow and tissue pO_2_ response to forced treadmill exercise in awake mice. To our knowledge, this is the first study performing both direct measure of brain tissue pO_2_ during acute forced exercise and underlying microvascular response at capillary and non-capillary levels. We observed that cerebral perfusion and oxygenation are enhanced during running at 5 m/min compared to rest. At faster running speeds (10 and 15 m/min), decreasing trends in arteriolar and capillary flow speed were observed, which could be due to cerebral autoregulation and constriction of arterioles in response to blood pressure increase. However, tissue pO_2_ was maintained, likely due to an increase in RBC linear density. Higher cerebral oxygenation at exercise levels 5–15 m/min suggests beneficial effects of exercise in situations where oxygen delivery to the brain is compromised, such as in aging, atherosclerosis and Alzheimer Disease.

## Introduction

Brain function is critically dependent on sufficient oxygen supply by cerebral vasculature because of its high oxygen metabolism rate and the lack of an oxygen reserve within the brain. Exercise induces changes in the cerebral metabolic rate of oxygen (CMRO_2_)^1–4^ and increases demand on the cardiovascular and pulmonary systems, with subsequent effects on cerebral vascular oxygenation and cognitive function^5–7^. While cerebral tissue pO_2_ at rest and during neural stimulation has been the subject of several studies in awake and anesthetized subjects^8–13^, the response of cerebral tissue pO_2_ to acute exercise has not been described.

Exercise-induced changes in several parameters that contribute to cerebral tissue oxygenation [such as the heat rate, cardiac output, blood pressure, blood pO_2_ and pCO_2_, CMRO_2_ and cerebral blood flow (CBF)] have been well characterized before^1,4,5,14–20^. However, due to the interplay between these parameters and possible redistribution of CBF in different brain regions during exercise, cerebral tissue pO_2_ response to exercise cannot be predicted reliably using the available data. There are also a number of near-infrared spectroscopy (NIRS) studies that investigated the oxygen saturation of hemoglobin in the brain during exercise^1,5,7,15,21^, but again the interpretation of tissue oxygenation from these data is not straightforward, because cerebral tissue pO_2_ is affected by other factors, such as CBF and CMRO_2_, which are modulated by exercise. Only one study measured tissue pO_2_, using Clark-type electrodes, in the hippocampal region of rats during exercise (swimming)^22^. Despite many studies on whole cerebral blood flow changes during exercise^14,17–20^, the microvascular substrate of tissue oxygenation response is not well characterized.

In this study, using two-photon microscopy and the O_2_ sensitive two-photon enhanced phosphorescent dye PtP-C343^23^, we directly measured cerebral tissue pO_2_ and capillary flow in awake animals during acute exercise. Blood flow speed in larger vessels was also quantified using Doppler optical coherence tomography (Doppler OCT) to investigate how changes in capillary flow are mediated. To our knowledge, this is the first study investigating the relationship between the cerebral tissue pO_2_ (direct measure), exercise intensity and the underlying microvascular response involved.

## Results

### Animal training and awake imaging

**Figure 1:**
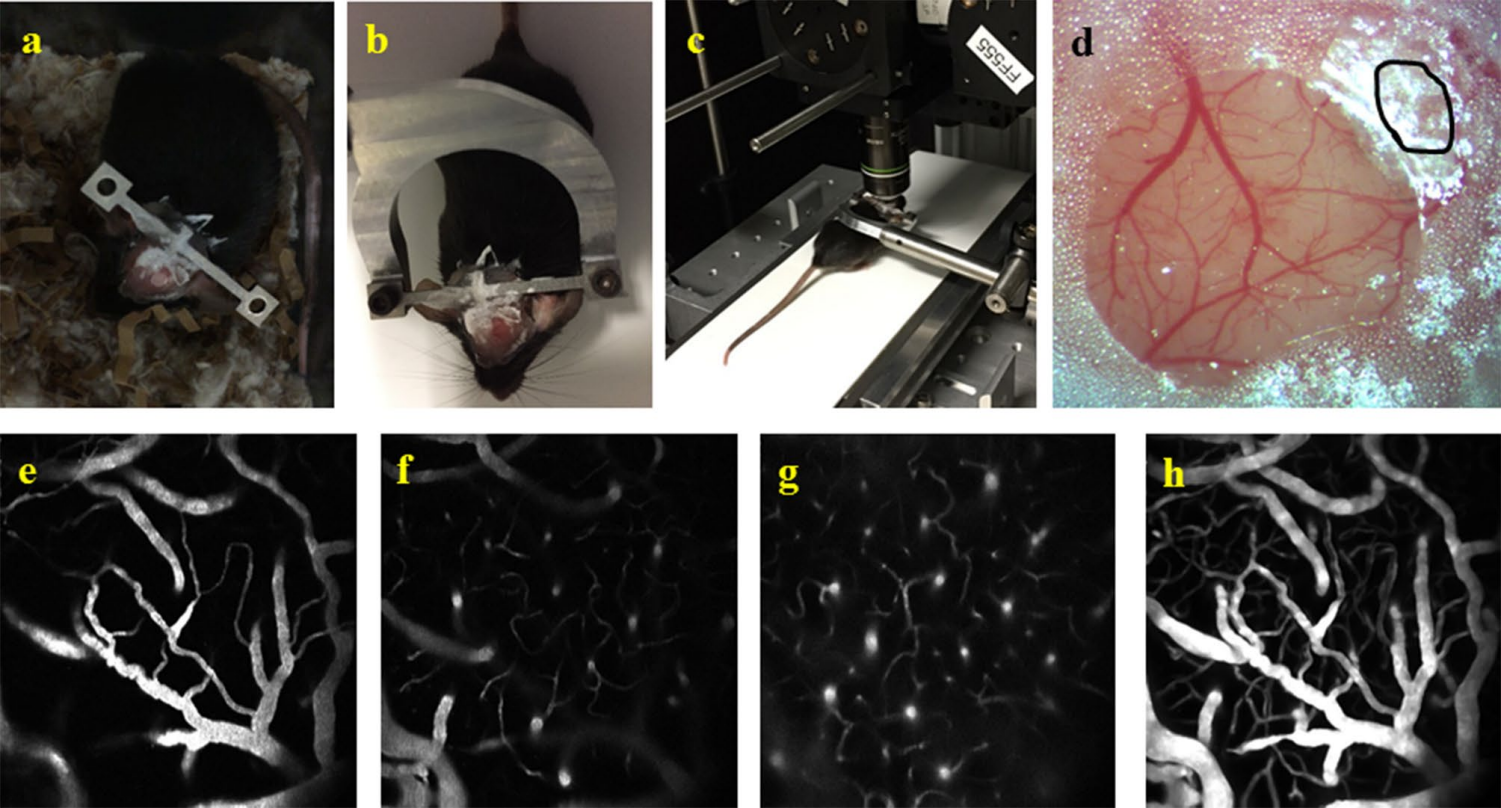
**(a)** A custom head-plate made from titanium was glued to the skull to allow animal restraint on the treadmill. **(b)** The implanted titanium bar was screwed to a holder to fix the animal’s head. **(c)** Restrained animal on the motorized treadmill under two-photon setup. **(d)** Thinned skull cranial window preparation with a small thinned region next to the coverslip uncovered by dental cement (marked with black lines) for pO_2_ dye injection. **(e–g)** Representative two-photon fluorescent images of microvasculature (600 µm × 600 µm) at depths of ~ 20, 50 and 120 μm. **(h)***En face* maximum intensity projection (MIP) of the 3D microvascular angiogram through the depths 0–150 μm.

Animals were implanted with titanium headposts for fixation on a motor-driven treadmill for measurements at fixed running speeds (Fig. 1a–c). A reinforced thinned-skull window^24^ was created for brain imaging (Fig. 1d). Before starting the main measurements, animals were trained on the treadmill over 5 days (see Table 1 for detailed protocol) to learn how to adjust their speed and also to habituate to the head restraint and minimize stress. On day 1 and day 2 of training, it was difficult for the animals to adjust to the treadmill speed. From day 3, animals showed improved running skill. By the end of last training session, animals were able to run very well, up to the running speed of 18–20 m/min (Supplementary video 1). Animals could also run properly on the treadmill when placed under imaging setups (Supplementary video 2). For two-photon measurements where the setup was enclosed by a dark chamber, a video baby monitor was used to observe the animal behavior in the dark and ensure that the animal is running properly during the imaging period.

**Table 1:** Training protocol.

Day 1	Morning	5 min At rest (0 m/min)	10 min 1 m/min → 2 m/min	
	Afternoon	10 min 1 m/min → 3 m/min		
Day 2	Morning	10 min 1 m/min → 2 m/min	1 min break	10 min 1 m/min → 4 m/min
	Afternoon	10 min 2 m/min → 4 m/min	1 min break	10 min 2 m/min → 5–6 m/min
Day 3	Morning	15 min 2 m/min → 5 m/min	2 min break	15 min 2 m/min → 7 m/min
	Afternoon	15 min 3 m/min → 7–8 m/min	2 min break	15 min 3 m/min → 10 m/min
Day 4	Morning	20 min 3 m/min → 7 m/min	3 min break	20 min 3 m/min → 11–12 m/min
	Afternoon	20 min 4 m/min → 12 m/min	3 min break	20 min 4 m/min → 16 m/min
Day 5	Morning	20 min 4 m/min → 15 m/min	3 min break	20 min 4 m/min → 18 m/min

During each training session, treadmill speed was gradually increased up to the maximum speed mentioned. From Day 2, each training session included two running blocks with 1–3 min break in between.

### Capillary blood flow

**Figure 2:**
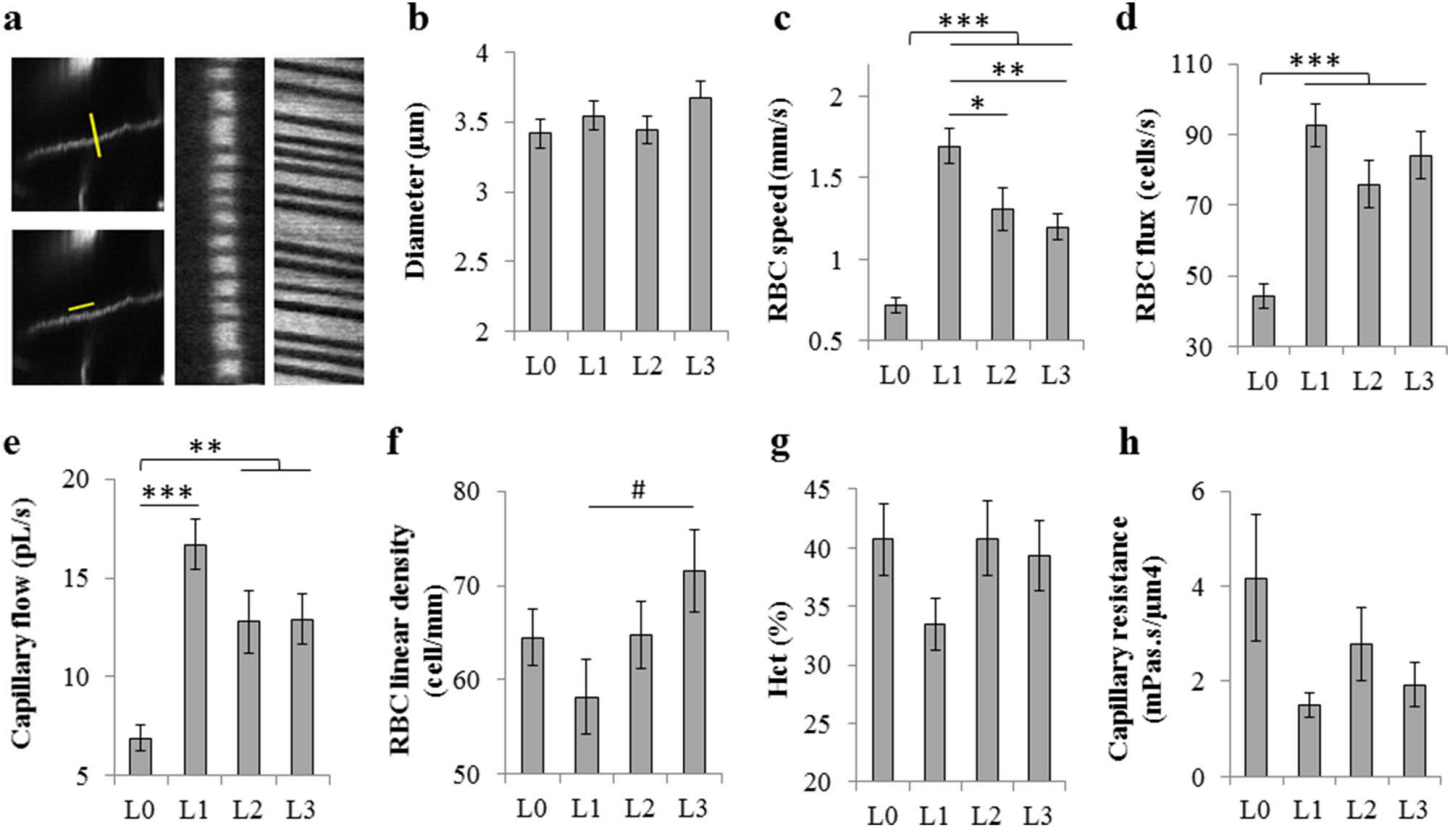
Capillary blood flow. **(a)** Perpendicular (upper left) and longitudinal (lower left) scans over selected capillaries and obtained space–time image (middle: perpendicular scan; right: longitudinal scan), from which capillary flow parameters were calculated. (**b**–**h**) Capillary diameter (**b**), RBC speed (**c**) and RBC flux (**d**) were obtained from space–time images. Capillary volumetric flow (**e**), RBC linear density (**f**), capillary hematocrit (**g**) and capillary resistance (**h**) were estimated from diameter, flux and speed. (L0, n = 39; L1, n = 25; L2, n = 30; L3, n = 32 capillaries). L0: rest, L1: exercise level 1 (5 m/min), L2: exercise level 2 (10 m/min), L3: exercise level 3 (15 m/min). Results are presented as mean ± s.e.m. Statistical significance was calculated using ANOVA followed by Tukey HSD post hoc test. ***p < 0.001, **p < 0.01, *p < 0.05, #p-value approaches significance (p < 0.1).

Capillary flow parameters were measured at rest (L0) and three treadmill speeds of 5 (L1), 10 (L2) and 15 m/min (L3), using a custom-built two-photon laser-scanning microscope^9^. Brain movement was minimal when the animal was restrained on the treadmill, which allowed imaging of clear angiograms (Fig. 1e–h). For each animal, a region of interest (600 µm × 600 µm) was chosen and capillary flow parameters were measured for ~ 10 capillaries (diameter < 10 µm) from longitudinal and perpendicular line-scans (Fig. 2a). With our fixation setup, capillary movement was minimal at different treadmill speeds which allowed reliable capillary flow imaging with line scans. Representative examples of the space–time images from two-photon longitudinal and perpendicular line scans at the treadmill speed of 15 m/min are shown in Supplementary videos 3–6.

Capillary blood flow imaging revealed a dramatic increase in RBC speed from L0 to L1 (135%, p < 0.001). With further increase in treadmill speed, a slight decrease in RBC speed was observed (p < 0.05), although RBC speeds at L2 and L3 remained higher than L0 (p < 0.001) (Fig. 2c). RBC flux and capillary flow also generally followed the same trend (Fig. 2d, e). On the other hand, there were no significant changes in capillary diameter (Fig. 2b) with the exercise level. However, RBC linear density showed an increasing trend from L1 to L3, which approached statistical significance for L3 versus L1 (p = 0.08, Fig. 2f). Capillary hematocrit values were in agreement with previous reports^8,9^, but no significant trend was observed with exercise intensity (Fig. 2g). This is not surprising, because hematocrit was extracted from capillary diameter, RBC speed, RBC speed and assuming a value for RBC volume (55 μm^3^ for C57Bl/6 mice^25^), with some assumptions. Both indirect estimation from several parameters and the assumptions made, lead to more scattering in hematocrit data. Calculated resistance to capillary flow showed a decreasing trend during exercise (L0 versus exercise levels 1–3), but did not reach statistical significance (p = 0.21 for L0 versus L1, Fig. 2h). This decreasing trend was in agreement with increased capillary flow during exercise (compared to rest).

### Non-capillary blood flow

**Figure 3:**
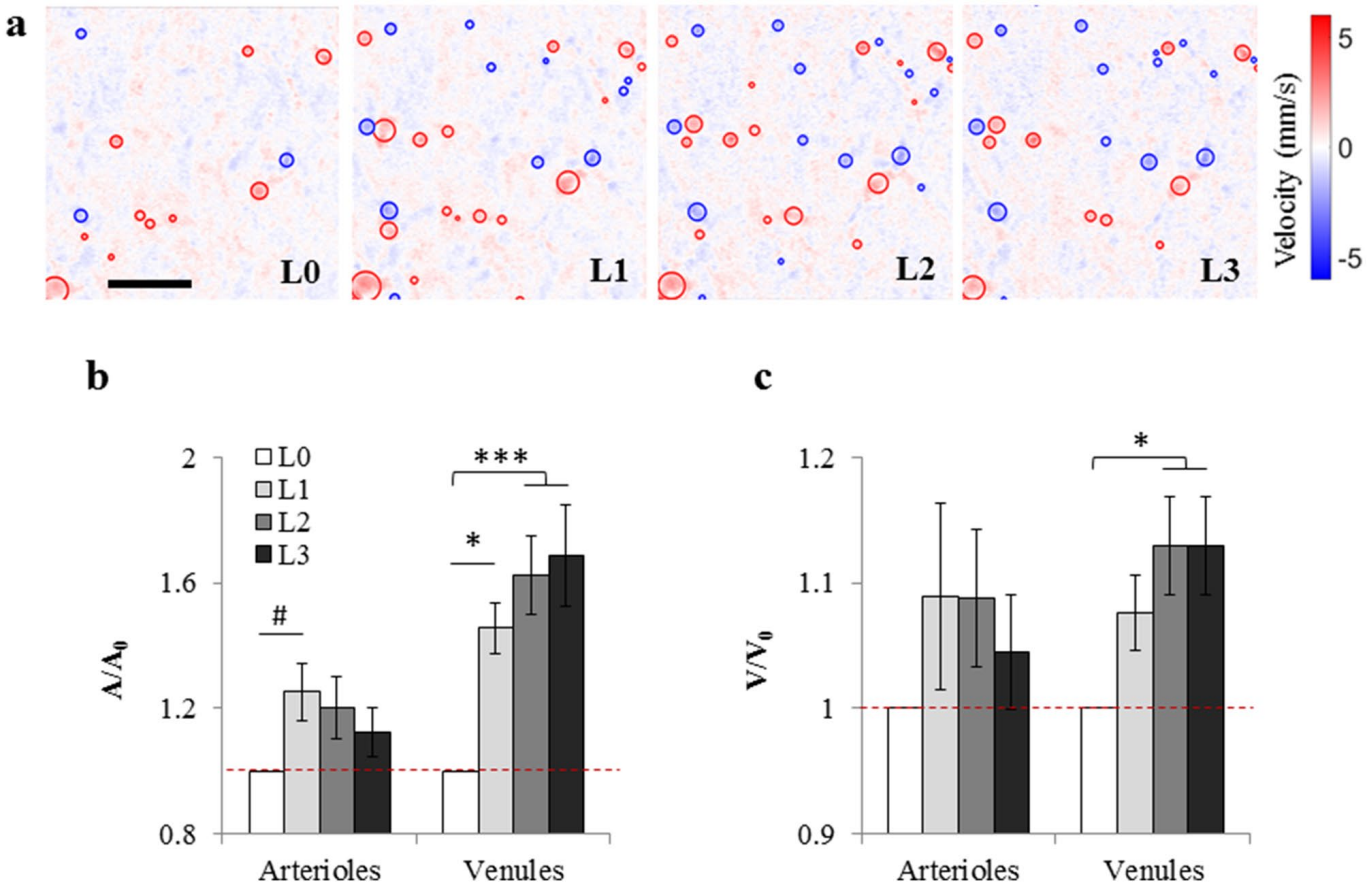
Non-capillary blood flow. **(a)** Representative *en face* slices through the OCT Doppler-velocity volume at a cortical depth of approximately 100 μm for exercise levels L0 to L3. The scale bar is 200 µm. Positive velocity (red) represents downward flow (arterioles) and negative velocity (blue) represents upward flow (venules). For each slice in the OCT velocity volume, arterioles and venules were detected (red and blue circles) and their area and average velocity were obtained. Same vessels were identified on OCT velocity volumes from different exercise levels and the variation of these parameters with the exercise level was quantified. **(b, c)** Average vessel cross-sectional area **(b)** and blood velocity **(c)** of individual arterioles and venules. For each vessel, parameters were normalized to the values at rest. The dashed lines represent the rest (arterioles: n = 23 vessels; venules: n = 22 vessels). L0: rest, L1: exercise level 1 (5 m/min), L2: exercise level 2 (10 m/min), L3: exercise level 3 (15 m/min). Bar plots represent mean ± s.e.m. Statistical significance was calculated using ANOVA followed by Tukey HSD post hoc test. ***p < 0.001, *p < 0.05, ^#^p-value approaches significance (p < 0.1).

We also measured the blood flow speed in non-capillary vessels (diameter > 10 μm) at rest (L0) and at three treadmill speeds of 5 (L1), 10 (L2) and 15 m/min (L3), using a custom optical coherence tomography (OCT) setup^9^. At each exercise levels, a 3D Doppler OCT volume was acquired over the exact same region (Fig. 3a). For each animal, a number of arterioles and venules were labelled and averaged (over depth). For vessels that were clearly identifiable across the all exercise levels, vessel cross-sectional area and blood velocity were obtained at exercise levels L0–L3.

As expected, the vessel size (cross sectional area) in both arterioles and venules was higher at L1 compared to the resting state, L0 (Fig. 3b). At L1, arterioles dilated by 25.4 ± 9.1% (p = 0.10, approaching significance) and venules dilated by 45.6 ± 8.2% (p = 0.023). Blood velocity also tended to be higher in both arterioles and venules at L1 compared to L0, but the differences did not reach statistical significance (p = 0.61 for arterioles and p = 0.33 for venules) (Fig. 3c). For venules, however, blood velocity was significantly higher at both L2 and L3 compared to L0 (p = 0.02 for both).

From L1 to L3, increasing or decreasing trends were observed in these parameters, and interestingly, the trends were in opposite directions in arterioles and venules. The vessel size showed a decreasing trend from L1 to L3 in arterioles, but an increasing trend was observed in venules (Fig. 3b). The same was true for blood velocity (Fig. 3c). For the vessel size, trend analysis (linear regression) from L1 to L3 showed a negative slope of -0.013 ± 0.013 min/m for arterioles, but a positive slope of + 0.023 ± 0.018 min/m for venules. The slopes were statistically different with a 76% confidence level. For blood velocity also a negative slope of -0.004 ± 0.008 min/m was observed for arterioles, while a positive slope of + 0.005 ± 0.005 min/m was found for venules, although the slopes were statistically different only with a 55% confidence level.

### Tissue pO_2_

**Figure 4:**
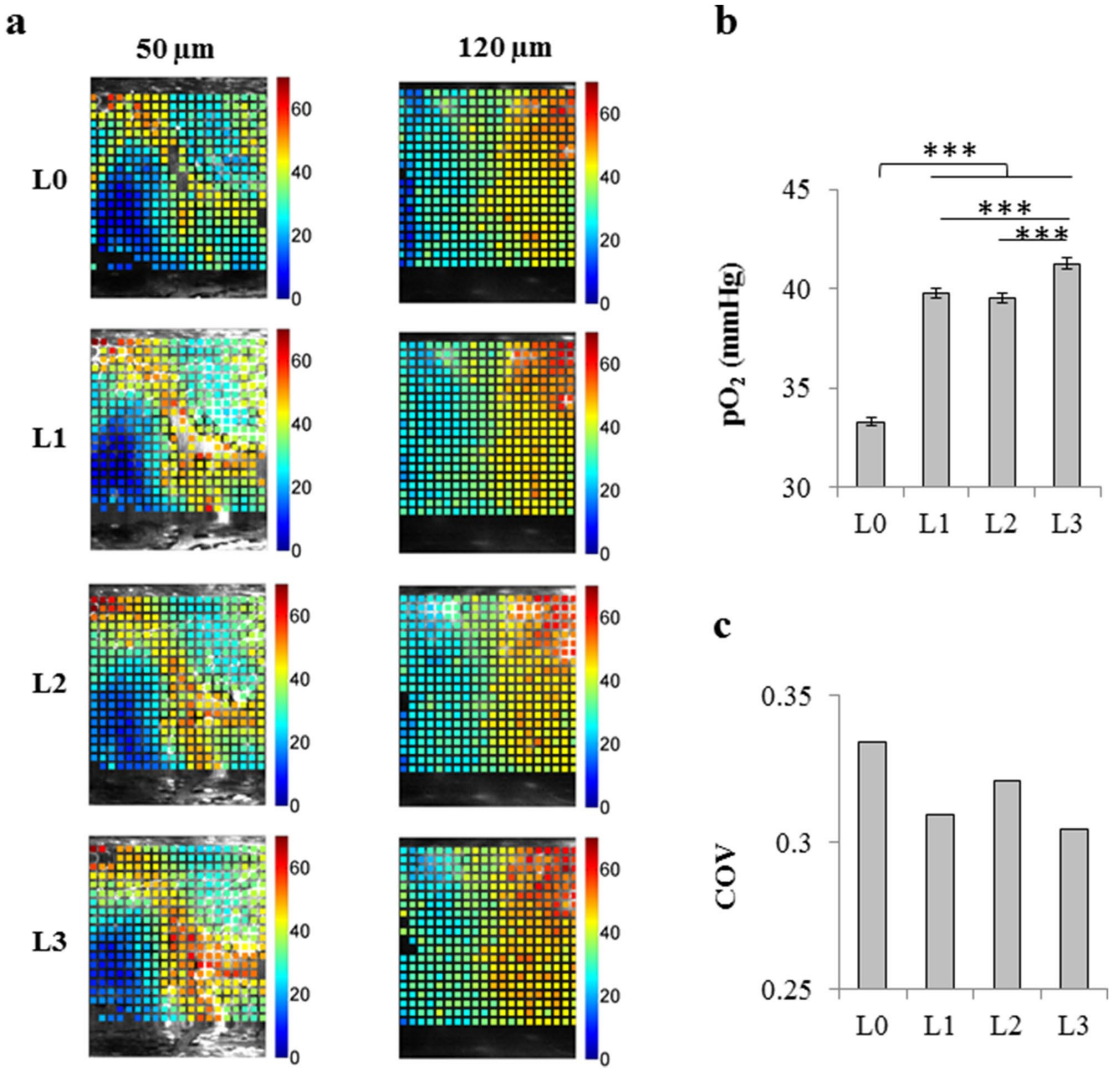
Cerebral tissue oxygenation. **(a)** Representative pO_2_ grids over 400 μm × 400 μm regions at the depth of 50 μm (left) or 120 μm (right) at different exercise levels (L0 to L3). Color bars show tissue pO_2_ in mmHg. **(b)** Average tissue pO_2_ at exercise levels L0 to L3. (L0, n = 3,089; L1, n = 2,838; L2, n = 3,002; L3, n = 2,431 sampled points). The bars represent mean ± s.e.m. Statistical significance was calculated using ANOVA followed by Tukey HSD post hoc test. ***p < 0.001. **(c)** Spatial heterogeneity of pO_2_ distribution in tissue (defined as coefficient of variation = SD/mean) for exercise levels L0 to L3. L0: rest, L1: exercise level 1 (5 m/min), L2: exercise level 2 (10 m/min), L3: exercise level 3 (15 m/min).

Tissue pO_2_ imaging was performed using our custom-built laser scanning microscope^9^ and the O_2_− sensitive phosphorescent dye PtP-C343^23^. A 400 μm × 400 μm region was chosen and grid measurements were performed at three depths (40–50 μm intervals, up to ~ 150 μm deep) (Fig. 4a) at rest (L0) and at the treadmill speeds of 5 (L1), 10 (L2) and 15 m/min (L3).

A significant increase in tissue pO_2_ was observed from L0 to L1 (from 33.3 ± 0.2 to 39.7 ± 0.2 mmHg, 19.3% increase, p < 0.001). With further increase in the treadmill speed, the changes in tissue pO_2_ were small. There was no change between L1 and L2 (39.7 ± 0.2 and 39.5 ± 0.2 mmHg, respectively) and tissue pO_2_ only increased to 41.3 ± 0.2 mmHg at L3 (p < 0.001) (Fig. 4b). Tissue pO_2_ spatial distribution tended to be more homogeneous during treadmill exercise compared with the resting state (Fig. 4c).

### Breathing rate

**Figure 5:**
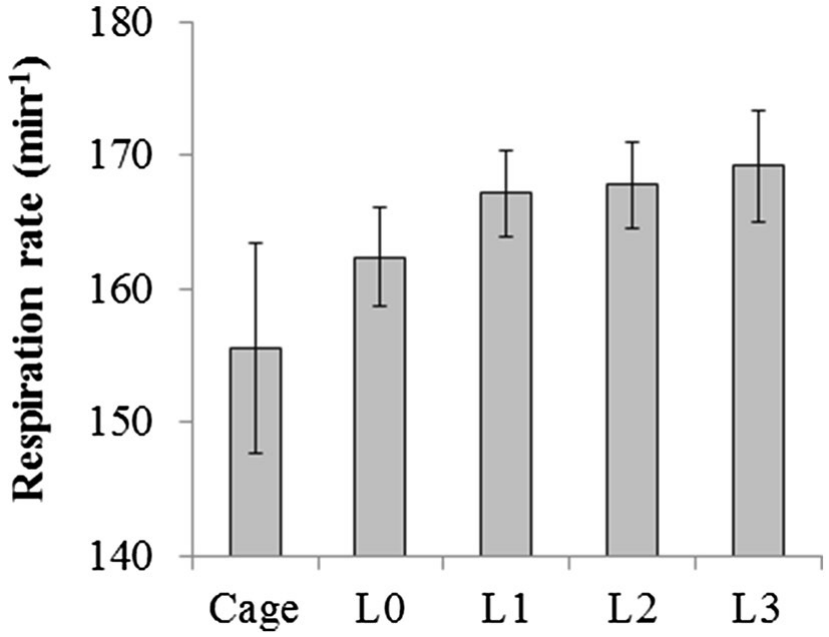
Breathing rate of mice in the cage or while fixed on the treadmill at exercise levels L0 to L3 (cage, n = 13; L0, n = 14; L1, n = 14; L2, n = 12; L3, n = 13). Breathing rate was weakly correlated with the exercise level (p = 0.26). L0: rest, L1: exercise level 1 (5 m/min), L2: exercise level 2 (10 m/min), L3: exercise level 3 (15 m/min).

As supplementary data, the breathing rate of the mice was also monitored using a mmWave sensing device. For each imaging session (capillary flow, non-capillary flow and tissue pO_2_), breathing rate was measured when the animal was in the cage, on the treadmill but at rest (L0) and right after the sessions at running speeds of 5 (L1), 10 (L2) and 15 m/min (L3). No measurement was obtained during the treadmill running because of the movement noise. Breathing rate after each exercise session was weakly correlated with the exercise intensity (p = 0.26) (Fig. 5).

## Discussion

In this study we examined cerebral blood flow and tissue pO_2_ response to forced treadmill exercise in healthy young mice. To our knowledge, this is the first study performing both direct measure of tissue oxygenation during acute forced exercise and underlying microvascular response at capillary and non-capillary levels.

### Enhanced cerebral perfusion and oxygenation during exercise compared with rest

While some studies report that global CBF remains relatively stable by exercise, based on several studies using a range of methods, it is now generally accepted that regional CBF increases during mild to moderate exercise^1,17,19,20,26,27^. Our data also indicates that during forced running at 5 m/min, for both arterioles and venules, vessels dilated and blood velocity tended to increase (Fig. 3b, c), compared to rest. Capillary blood flow, RBC speed and RBC flux were also increased from L0 to L1 (Fig. 2c–e).

Although not measured here, it has been shown that the exercise-driven increases in heart rate and cardiac output increase the arterial blood pressure (BP)^5,20^. However, arterial BP remains within the cerebral autoregulation range. Therefore, the increase in CBF is potentially largely due to the vasodilatory effect of CO_2_ (because of higher systemic CO_2_ during exercise^20,28^ and also local CO_2_ increase by enhanced cerebral metabolism^14^) in the presence of increased arterial BP^1,4,5,14,17,26,27^. Cerebral perfusion is governed by arterial BP and cerebrovascular resistance (CVR), assuming that venous blood pressure remains roughly constant. Since arterial BP increases slightly during exercise and CVR decreases (dilated vessels, Fig. 3b), it is expected that blood flow increases during exercise compared to the rest (Fig. 3c).

During exercise, capillary resistance showed a decreasing trend, but the decrease was not statistically significant (Fig. 2h). Because capillary resistance is not measured directly, but estimated from other capillary parameters (diameter, RBC speed, RBC flux, and RBC volume), it is not surprising that the capillary resistance data is more scattered and we do not see a clear reduction, as potentially expected. Taking into account that capillaries did not dilate (Fig. 2b), it is very likely that increased pressure gradient across the capillary bed also plays a role in higher capillary blood flow during exercise (Fig. 2e). Assuming a constant venous BP, this could be achieved by a systemic increase in arterial BP (not measured here, but expected from previous studies) and vasodilation of upstream arterioles (Fig. 3b) which reduces the resistance to flow upstream and hence increases the inlet capillary pressure.

Elevated tissue pO_2_ during exercise (Fig. 4b) indicates that although cerebral metabolic activity is potentially increased^14^, the increase in cerebral perfusion is much larger than enhanced cerebral metabolic demand for O_2_. Capillaries were not dilated during exercise, but the increases in the speed and flux of RBCs enhanced the tissue pO_2_, with a tissue oxygenation that tended to be more homogeneous when mice were running (Fig. 4). This observation is in agreement with NIRS studies in human showing increased cerebral blood oxygenation with moderate or hard exercise^1,5,7,15,21^.

### Autoregulation tends to lower the cerebral blood flow speed with increasing running speed?

With increasing treadmill speed from 5 m/min to 15 m/min, flow speed of blood was decreased in capillaries and decreasing trends were observed in arterioles (Figs. 2 and 3c). This similar trend in capillaries and arterioles suggests that during exercise, capillary blood flow is largely mediated by arterioles. The decrease in arteriolar blood flow speed was associated with a constriction of arterioles with increased running speed (Fig. 3b). On the other hand, for venules the reverse trends were observed (vessel dilation and increased blood velocity with increasing exercise intensity) (Fig. 3b, c).

Many studies have reported that while whole cerebral blood flow is increased during moderate and high intensity dynamic exercise as a function of exercise intensity, it decreases by extreme intensity exercise (oxygen uptake > 60% of maximal oxygen uptake)^1,17,19,22,26,27^. It is stated that this reduction in CBF is due to hyperventilation-induced decrease in partial pressure of arterial CO_2_ (p_a_CO_2_) during intense exercise and consequent vasoconstriction of cerebral arteries^1,7,15,16,27^. Our observation of an increasing trend of blood flow speed in arterioles and capillaries from rest to L1 and a decreasing trend afterwards (Figs. 2c,e, 3c) is consistent with those reports. We also found that arterioles dilate from L0 to L1, but then tend to constrict as exercise intensity increases from L1 to L3 (Fig. 3b), again consistent with above hypothesis.

Previously, it has been shown in 8-week-old male C57BL/6J mice that maximal oxygen uptake (VO_2,max_) is reached at a treadmill speed of ~ 20 m/min for sedentary (untrained) mice and ~ 22 m/min after 1 week of training. In these experiments, however, the treadmill was 25° inclined^29,30^. In another study with 12-month-old male C57BL/6J mice and a treadmill inclination of 0° (similar to us), VO_2,max_ was reached at a running speed of ~ 25 m/min for untrained animals^31^. We did not measure the oxygen uptake (VO_2_) in our experiments, but using data from Schefer and Talan (1996)^31^, VO_2_ can be roughly estimated as ~ 60%, 70% and 80% of VO_2,max_ at treadmill speeds of 5,10, and 15 m/min, respectively; while at rest, VO_2_ is nearly 30% of VO_2,max_. Therefore, based on above discussion, in our experiments decreased p_a_CO_2_ at higher exercise intensities (not measured here) could contribute to the observed arteriolar vasoconstriction (Fig. 3b) and reduced capillary blood flow (Fig. 2e) from L1 to L3.

Although decreased p_a_CO_2_ could be involved in constriction of arterioles with increasing treadmill speed, but the contrast of arterioles constricting and venules dilating (Fig. 3b, c) could also be a measure of autoregulation: With increased arterial BP with exercise intensity (not measured here, but based on previous data^4^), arterioles will constrict to maintain a constant CBF. Decreased vessel size, increases the CVR and reduces the blood flow in arterioles. Despite increased CVR, there will remain a slight increase of blood pressure and thus, veins which are passive will dilate. In fact, it has been previously shown that dynamic cerebral autoregulation remains stable during physical challenge in healthy persons^26^. Therefore, it seems that autoregulation plays a role here, but since we did not measure the arterial BP for each condition, we are not measuring autoregulation curves and further studies are needed to confirm this hypothesis.

### Regulatory mechanisms at the capillary level maintain tissue pO_2_ despite decreasing perfusion with increasing running speed

The trend we observed in tissue pO_2_ with exercise intensity is in agreement with previous NIRS studies in healthy subjects, showing the existence of a quadratic relationship between cerebral oxygenated hemoglobin and exercise intensity, rising between moderate and hard intensities, then falling at very hard intensities^1,7,15,21^. It has been reported that global CMRO_2_ is unchanged by the transition from rest to moderate intense exercise, while it is enhanced by more vigorous exercise^4,14,28^. Regional CMRO_2_ in some brain regions such as motor and sensory area, however, increases even during mild exercise^14^. The increased tissue oxygenation at L1 (compared to rest) (Fig. 4b) implies more increase in cerebral perfusion than increased CMRO_2_. With increased exercise intensity, however, tissue pO_2_ did not change greatly (Fig. 4b). This implies that the new balance between the oxygen supply and consumption is maintained from L1 to L3, but from our data it is not possible to conclude how exactly oxygen delivery to tissue and CMRO_2_ change individually.

Surprisingly, despite decreasing trends in arteriolar and capillary blood flow speeds and constant capillary diameter from L1 to L3 (Figs. 2 and 3b, c), tissue pO_2_ was not decreased (Fig. 4). One interesting finding was that from L1 to L3, RBC speed slightly decreased, while RBC flux was almost unchanged (Fig. 2c, d). This led to increased RBC linear density (Fig. 2f), which could explain the maintained tissue oxygenation despite decreased perfusion. The increase in RBC linear density could be explained by a fluid shift between capillaries and interstitial space during exercise due to the accumulation of osmotically active metabolites and filtration as a consequence of increased capillary pressure^16,32^. The rigidification of RBCs during exercise^32–35^ may also hinder the capillary perfusion and play a role in reduced RBC speed and increased RBC linear density in capillaries.

The observation that cerebral tissue is more oxygenated at exercise levels of 5–15 m/min suggests beneficial effects of exercise in situations that oxygen delivery to the brain is compromised, such as in aging^9^, atherosclerosis^36–38^, hypertension^39^ and Alzheimer Disease (AD)^40^. Although very short-term effects of acute exercise were investigated in this study, but it is expected that routine long-term exercise could improve cerebral oxygenation and function in above conditions. In fact, previous studies indicate the influence of prolonged exercise routines in maintaining cognitive health. For example, we have previously shown in mice that AD is associated with hemodynamic alterations, lower microvascular density and brain oxygenation disruption, but these alterations were reversed by long-term (3-months) voluntary exercise^40–42^. Others have shown that regular exercise enhances cognitive performance in humans^43–45^ and improves the vascular function in a mouse model of atherosclerosis^46^. Oxidative stress associated with aging has also been found to be reduced with exercise^47,48^.

## Limitations and future perspectives

We did not measure the oxygen uptake (VO_2_) during the experiments. However, previous studies with C57BL/6J mice confirmed almost linear increase in VO_2_ with running speed in the range of 3–20 m/min on the treadmill^29–31^, implying that running speed can be a proxy of exercise intensity in this range. Although breathing rate alone is not an accurate predictor of VO_2_, our breathing rate measurements also showed a weak correlation with the running speed (Fig. 5). As discussed above, using literature data for untrained male C57BL/6J mice^31^, it was estimated that VO_2,max_ is reached at the treadmill speed of ~ 25 m/min and that the treadmill speeds of 5, 10, and 15 m/min used here are roughly equivalent to ~ 60, 70, and 80% of VO_2,max_. Therefore, the exercise intensities used in our study were below the maximal oxygen uptake rate and exhaustion level. Monitoring of animals during the imaging sessions also confirmed that they did not run to exhaustion.

In designing the experimental protocols, the running speeds (5, 10 and 15 m/min) and the duration of each running session (~ 20 min) were chosen such that the animals do not experience exhaustion. In addition, a 10 min break was given between incremental running sessions to minimize fatigue accumulation and exhaustion of the animals. However, fatigue may still accumulate during incremental running sessions which can affect the physiological parameters and the measurements at higher treadmill speeds. For example, it would be interesting to see how cerebral tissue pO_2_ changes with time during each 20 min running session, or how different would be the results if there was a bigger time interval between running sessions to ensure complete animal recovery. Considering the time needed for pO_2_ measurement at each sampling point and our priority to scan an adequately large area at each running speed, in these experiments we were not able to measure pO_2_ changes with time at each running speed. However, it would a valuable information and could be done in future studies. In addition, one could do a different experimental design in which measurements at different running speeds are done with a longer break time between incremental treadmill speeds. Even one may decide to have only one running session per day with a specific treadmill speed to completely remove the confounding effects of fatigue accumulation. However, in any design in which animals need to be removed from the imaging setup between incremental running speeds, it would be difficult to ensure repeated measurements on the exact same regions (as was performed in this study). Further detailed studied and also development of appropriate mathematical models are demanding in order to elucidate the underlying mechanisms involved in cerebral response during exercise and probably to optimize the exercise routines to achieve the most beneficial effects.

## Conclusion

In this study, we examined the cerebral tissue pO_2_ response to forced treadmill exercise and investigated the underlying vascular substrate in healthy young mice. Running at 5 m/min enhanced the cerebral perfusion and oxygenation compared to rest. At faster running speeds (10 and 15 m/min), a decreasing trend in arteriolar and capillary flow speed was observed, which could be due to cerebral autoregulation in response to blood pressure increase. However, in spite of this decreased perfusion, tissue pO_2_ was maintained, likely due to an increase in RBC linear density. These findings can enhance our understanding of the nature of the relationship between exercise intensity and brain function.

## Materials and methods

### Animal preparation

Animal handling and surgical procedures were approved by the ethics committee of the research center of the Montreal Heart Institute. All experiments were performed in accordance with the ARRIVE guidelines and the recommendations of the Canadian Council on Animal Care. Six male C57/BL6 mice (8 weeks old, 19–24 g weight) were housed in 12-h light/dark cycle until imaging. 8–10 days before measurements, a thinned skull window was created over the left barrel cortex under isoflurane anesthesia (2.0% in pure oxygen), using the method developed by Shih et al.^24^ with some modifications as described in our previous paper^9^. Briefly, the scalp was removed and the exposed skull was covered with a thin layer of tissue adhesive (Vetbond). A custom head-plate made from titanium was fixed on the skull using dental cement (Fig. 1a). The skull was then slowly thinned to translucency with a micro-drill (OmniDrill 35, World Precision, USA). A 150 μm-thick cover glass was glued to the dried window using cyanoacrylate glue and the edges were sealed with dental cement to form a 3 mm diameter cranial window. A small thinned region at the edge of the cover glass (~ 0.5 mm) was left uncovered with dental cement (Fig. 1d) to allow the injection of the PtP-C343 dye into tissue through the soft thinned membrane. During the surgery, animals were fixed on a controlled physiological monitoring system (LabeoTech, Canada) which enabled continuous monitoring of the rectal temperature, respiration and heart rate. Ketoprofen (s.c., 5 mg/kg, Merial, Canada) and buprenorphine (s.c., 0.05 mg/kg, Reckitt Benckiser Healthcare, UK) were injected before the surgery and baytril (i.p., 5 mg/kg, Bayer, Germany) was injected after the surgery. Injections were repeated 24 h after the surgery.

### Animal training and awake imaging

A motor-driven treadmill (Mini-Mover, LP series, USA) was used to incite the animals to run at fixed speeds. The implanted titanium bar was screwed to a holder, which fixed the animal’s head, but the limbs were free to move (Fig. 1b, c). Before starting the main measurements, animals were trained on the treadmill to learn how to adjust their speed and also to habituate to the head restraint and minimize stress. Training sessions were started 3–4 days after the surgery to ensure animal recovery after the surgery. The animals were trained two times per day (in the morning and in the afternoon) for 4 days with gradual increase in the duration of restraint and the maximum treadmill speed. On the fifth day, one more training session was performed in the morning and in the afternoon capillary blood flow imaging was performed, followed by the Doppler OCT and pO_2_ imaging in the next day (in the morning and afternoon, respectively). Table 1 shows the detail of the training protocol.

### Capillary blood flow imaging with two-photon laser-scanning fluorescence microscopy

Capillary flow parameters were measured using a custom-built two-photon laser-scanning microscope described before^9^. 400 mg/kg of 2MDa dextran-FITC (50 mg/ml in saline, Sigma) was injected through the tail vein. Due to the injected fluorescent dye, the plasma appeared bright in the images while RBCs appeared as dark shadows. A region of interest (600 µm × 600 µm) was chosen and capillary flow parameters were measured for ~ 10 capillaries (7–13 capillaries, diameter < 10 µm) at the depth of 100–300 µm at rest (L0) and at three treadmill speeds of 5 (L1), 10 (L2) and 15 m/min (L3). Measurements at each exercise level lasted for 20 min. After each exercise level (except rest, L0), a 10 min break and water was given.

For each capillary, longitudinal and perpendicular line-scans measuring 100 points along a straight line (~ 20 μm long) were performed at a line-rate of 800 Hz. The line-scans were performed continuously and 250 ms segments of the line-scan data (200 lines) were used to create space–time images with dark streaks due to the motion of red blood cells (RBCs) (Fig. 2a). The space–time images were used to obtain the following parameters for each capillary, as described before^8,9^: internal diameter, RBC velocity; capillary volumetric flow, RBC flux (cells/s), hematocrit and capillary resistance. RBC linear density was also calculated as flux/speed.

### Non-capillary blood flow measurements with Doppler-OCT

Blood flow speed in non-capillary vessels (diameter > 10 μm) was measured at rest (L0) and at three treadmill speeds of 5 (L1), 10 (L2) and 15 m/min (L3), using a custom optical coherence tomography (OCT) setup, as described by Moeini et al. (2018)^9^. At each exercise levels, a 3D Doppler OCT volume was acquired over a cortical surface of ~ 700 μm × 700 μm and processed with the procedure explained in Moeini et al. (2018)^9^ (Fig. 3a). For each animal, a number of arterioles and venules were labelled and averaged (over depth) cross-sectional area and blood velocity of these vessels were obtained at exercise levels L0–L3. Each exercise level lasted for 20 min, during which a Doppler scan was performed (started at 10 min). After each exercise level (except rest, L0), a 10 min break was given. OCT scans at different exercise intensities were performed over the exact same region.

### Tissue pO_2_ imaging by two-photon phosphorescence lifetime microscopy

Tissue pO_2_ imaging was performed using the custom-built laser scanning microscope and the O_2_− sensitive phosphorescent dye PtP-C343^23^, as described before^9^. The PtP-C343 dye solution (~ 150 μM in ACSF) was slowly injected into the brain tissue ~ 300 μm below the surface with a glass micropipette using a microsyringe injector (UMP3, World Precision Instruments, USA). The injection was done through the thinned skull next to the cover glass. The diffusion of the injected dye to the surrounding tissue allowed imaging of cerebral tissue beneath the cover glass. ~ 200 μl 70 KDa Rhodamine B isothiocyanate-Dextran (20 mg/ml in saline, Sigma) was also injected through the tail vein to visualize the vasculature. A 400 μm × 400 μm region was chosen and grid measurements (400 points) were performed at three depths (40–50 μm intervals, up to ~ 150 μm deep) (Fig. 4a) at rest (L0) and at the treadmill speeds of 5 (L1), 10 (L2) and 15 m/min (L3). Imaging at each exercise level lasted ~ 20 min. After each exercise level (except rest, L0), a 10 min break was given.

In grid measurements, 3,000 excitation cycles were averaged at each point. Each excitation cycle consisted of 25 μs excitation period in which the laser pulse was “on” followed by 275 μs “off” period in which the phosphorescence emission was allowed to decay^9^. Averaged phosphorescence decay at each point was fitted with a single-exponential curve to determine the phosphorescence lifetime. Tissue pO_2_ values were obtained from the lifetimes using a calibration curve. All sampling points at each exercise level were pooled to find the average tissue pO_2_ and spatial heterogeneity of tissue oxygenation (defined as SD/mean).

### Breathing rate

The breathing rate of the awake mice was monitored using a mmWave sensing device (IWR1443BOOST, Texas Instruments, USA) which operates based on Frequency Modulated Continuous Wave (FMCW) radar technology. In-house Matlab code was implemented to control the device and for data saving. The radar was placed 15 cm from the mice. Phase shifts over time was measured to obtain the chest displacement of the mice. Frequency analysis was performed to extract the breathing rate from the raw signal after high-pass filter (0.5 Hz) and phase unwrap.

For each imaging session (capillary flow, non-capillary flow and tissue pO_2_), breathing rate was measured when the animal was in the cage, on the treadmill but at rest (L0) and right after the sessions at running speeds of 5 (L1), 10 (L2) and 15 m/min (L3). No measurement was obtained during the treadmill running because of the movement noise. All data from different animals and different imaging sessions were pooled for each exercise level to find the average breathing rate at exercise intensities L0 to L3.

### Statistical analysis

The results are presented as mean ± s.e.m. Statistical significance was calculated using ANOVA followed by Tukey HSD post hoc test. Statistical significance was assigned at *p < 0.05, **p < 0.01, ***p < 0.001. The sample sizes were chosen empirically based on our previous experience.

In the case of non-capillary blood flow, linear regression was performed for trend analysis. Linear regression was performed for vessel size and blood velocity data at L1, L2 and L3 exercise intensity levels and the slope of the linear regression and the upper and lower confidence intervals were found. The confidence interval at which the slopes are different were then determined in each case.

## Supplementary information

Supplementary Information.

Supplementary Video 1.

Supplementary Video 2.

Supplementary Video 3.

Supplementary Video 4.

Supplementary Video 5.

Supplementary Video 6.

## Data Availability

The authors declare that the main part of data supporting the findings of this study is available within the paper and its supplementary figures. Data not presented within the paper or supplementary figures are available from the corresponding author upon reasonable request.
