# Supplementary material for: Cerebral tissue pO2 response to treadmill exercise in awake mice
Source: Sci Rep. 2020 Aug 7;10:13358. doi: 10.1038/s41598-020-70413-3 (PMC7414913; doi:10.1038/s41598-020-70413-3)
Supplement: Supplementary file 1 — Supplementary Information. [file 41598_2020_70413_MOESM1_ESM.pdf]

Supplementary Information

Cerebral tissue pO_2 response to treadmill exercise in awake mice

Mohammad Moeini[1,2], Christophe Cloutier-Tremblay[3], Xuecong Lu[2,3], Ashok Kakkar[4], Frédéric Lesage[2,3 *]

[1] Department of Biomedical Engineering, Amirkabir University of Technology (Tehran Polytechnic), Tehran, Iran

[2] Research Center of Montreal Heart Institute, Montréal, QC, Canada

[3] Biomedical Engineering Institute, École Polytechnique de Montréal, Montréal, QC, Canada

[4] Department of Chemistry, McGill University, Montréal, QC, Canada

Supplementary Video 1. Improvement of animal's running skills on the treadmill during the training sessions (2 sessions per day). The length of the time that mice were restrained and the maximum running speed were gradually increased each day.

Supplementary Video 2. After training, animals were able to run very well on the treadmill under imaging setups.

Supplementary Videos 3-6. Representative space-time images from two-photon longitudinal and perpendicular line scans of capillaries at the exercise level 3 (L3), showing minimal capillary movement and reliable measurements.
